# Digitise This! A Quick and Easy Remote Sensing Method to Monitor the Daily Extent of Dredge Plumes

**DOI:** 10.1371/journal.pone.0051668

**Published:** 2012-12-11

**Authors:** Richard D. Evans, Kathy L. Murray, Stuart N. Field, James A. Y. Moore, George Shedrawi, Barton G. Huntley, Peter Fearns, Mark Broomhall, Lachlan I. W. McKinna, Daniel Marrable

**Affiliations:** 1 Department of Environment and Conservation, Kensington, Western Australia, Australia; 2 Oceans Institute, University of Western Australia, Crawley, Western Australia, Australia; 3 Remote Sensing and Satellite Research Group, Department of Imaging and Applied Physics, Curtin University, Bentley, Western Australia, Australia; NASA Jet Propulsion Laboratory, United States of America

## Abstract

Technological advancements in remote sensing and GIS have improved natural resource managers’ abilities to monitor large-scale disturbances. In a time where many processes are heading towards automation, this study has regressed to simple techniques to bridge a gap found in the advancement of technology. The near-daily monitoring of dredge plume extent is common practice using Moderate Resolution Imaging Spectroradiometer (MODIS) imagery and associated algorithms to predict the total suspended solids (TSS) concentration in the surface waters originating from floods and dredge plumes. Unfortunately, these methods cannot determine the difference between dredge plume and benthic features in shallow, clear water. This case study at Barrow Island, Western Australia, uses hand digitising to demonstrate the ability of human interpretation to determine this difference with a level of confidence and compares the method to contemporary TSS methods. Hand digitising was quick, cheap and required very little training of staff to complete. Results of ANOSIM R statistics show remote sensing derived TSS provided similar spatial results if they were thresholded to at least 3 mg L^−1^. However, remote sensing derived TSS consistently provided false-positive readings of shallow benthic features as Plume with a threshold up to TSS of 6 mg L^−1^, and began providing false-negatives (excluding actual plume) at a threshold as low as 4 mg L^−1^. Semi-automated processes that estimate plume concentration and distinguish between plumes and shallow benthic features without the arbitrary nature of human interpretation would be preferred as a plume monitoring method. However, at this stage, the hand digitising method is very useful and is more accurate at determining plume boundaries over shallow benthic features and is accessible to all levels of management with basic training.

## Introduction

Industrialization, growing populations, and expansion of mining throughout the world, are impacting on natural coastal environments. Australia’s coastal environments are under increased pressure from development at present with enormous investment in the mining industry and the subsequent construction of harbours to service these ventures throughout Australia. Construction of pipelines, marinas and ports within these coastal environments often requires land reclamation and dredging of the sea floor with subsequent spoil disposal. The suspension of sediments due to these activities potentially has impacts on surrounding ecosystems, such as physical smothering of benthic communities, and decreased light availability which limits growth and metabolic processes of sea grass [1], algae [2], coral [3], [4] [5], [6] and larval fish [7]. Scientists and management agencies are under growing pressure to be able to understand the spatial extent and potential influences, particularly cumulative, of these activities within short time frames (daily) to enable appropriate response measures.

Projects that monitor environmental change are restricted by the availability of consecutive images (repeat capture schedule) over the required monitoring time period, and the spatial resolution available that is required to monitor the area of interest. Projects utilising high resolution imagery may be further limited by the cost and the time required to interpret such imagery with greater detail. Advances in technology and product availability, over the past two decades, has enabled satellite remote sensing products to become an accepted method for monitoring the spatial extent of terrestrial and aquatic impacts [8]–[12]. Imagery from satellite sensors such as Landsat Thematic Mapper (TM) and Enhanced Thematic Mapper+(ETM+) can be downloaded gratis and have a pixel resolution of 30×30 m. Landsat satellites have a repeat capture of every 16 days (see http://glovis.usgs.gov/), limiting the power to detect rapid temporal change. Other higher spatial resolution sensors such as Advanced Land Observing System’s Advanced Visible and Near Infrared Radiometer type 2 sensor (ALOS AVNIR-2), Quick Bird 2 (QB2) or World View 2 (WV2) (10×10 m, 2.5×2.5 m, and 2×2 m pixel resolution respectively) vary in regular repeat capture capability, ranging from four times per year to every couple of days, however the use of these sensors is cost prohibitive. When designing a monitoring project using remote sensing, an adaptive approach is required.

Imagery captured by the Moderate Resolution Imaging Spectroradiometer (MODIS) sensor is free to access and is well suited for monitoring daily events that occur on a wide spatial scale. The MODIS sensors, on board the Terra (EOS AM-1) and Aqua (EOS PM-1) satellites, achieve near-daily global coverage, capturing data across 36 spectral bands. MODIS data may be used in a number of ways ranging from observing atmospheric conditions to terrestrial and oceanic processes with pixel resolutions ranging from 1×1 km to 250×250 m pixels at nadir [13]. NASA provides MODIS imagery freely in raw format or as processed mosaiced georeferenced true colour enhanced imagery, making it very accessible for product generation or visual interpretation. Other studies have indicated MODIS band 1 (620–670 nm, centred on 645 nm) is a useful proxy for visualising elevated TSS loads in Australian marine waters [14], [15]. MODIS band 1 reflectance can be used to calculate L2 TSS products from regionally tuned empirical models to provide quantitative estimates of sediment load [16]–[18]. Similar approaches have been used for dredge plume monitoring in Australian waters by Islam et al. (2007) [19] where a TSS algorithm was developed for MODIS data during 2006 dredge operations at Hay Point, Queensland. However there are difficulties in applying Level 2 products in turbid and shallow waters. This can result very limited data quality or limited quantities of data where L2 processing fails. Hand digitising of MODIS imagery has also recently been used as a management tool for monitoring the spatial extent and distribution of sediment laden riverine flood plumes within the Great Barrier Reef World Heritage Area, Australia [15], [20].

Visual interpretation and hand digitization of either high resolution aerial photography or high, medium, or low resolution satellite imagery is a common method used for assessing environmental impacts in near-real time, as well as accessing historical imagery from archives to compare to targeted captures in the present. The hand digitising approach is a simple standard method adopted by some government agencies for monitoring, management and compliance in terrestrial environments: For example, in emergency fire digitising [21]–[23], deforestation [24], and mangrove growth and reduction [25], urban development [26], compliance prosecution cases for illegal land clearing [27] and over-flooding on Alaskan ice [28]. The visual interpretation of daily MODIS satellite imagery with manual digitization was considered to be a quick, relatively accurate and easy method to complement in-situ biological monitoring of coral reef communities and physical monitoring (sediment traps) of a dredge plume.

The objective of the digitisation monitoring method was to gain an understanding of the daily spatial extent and the temporal frequency dynamics of the plume (the sum of the plume’s daily presence). Analysis of the spatial/temporal frequency of the plume has the potential to highlight areas most affected, and create a better understanding of the influence this environmental change has on monitored sites of high biological significance. The results of the digitization method were compared to recognized semi-automated methods, using calibrated MODIS imagery to produce a total suspended solids (TSS) product. This paper highlights the strengths and weaknesses of each technique.

## Methods

### Location and Background of Study Area

**Figure 1:**
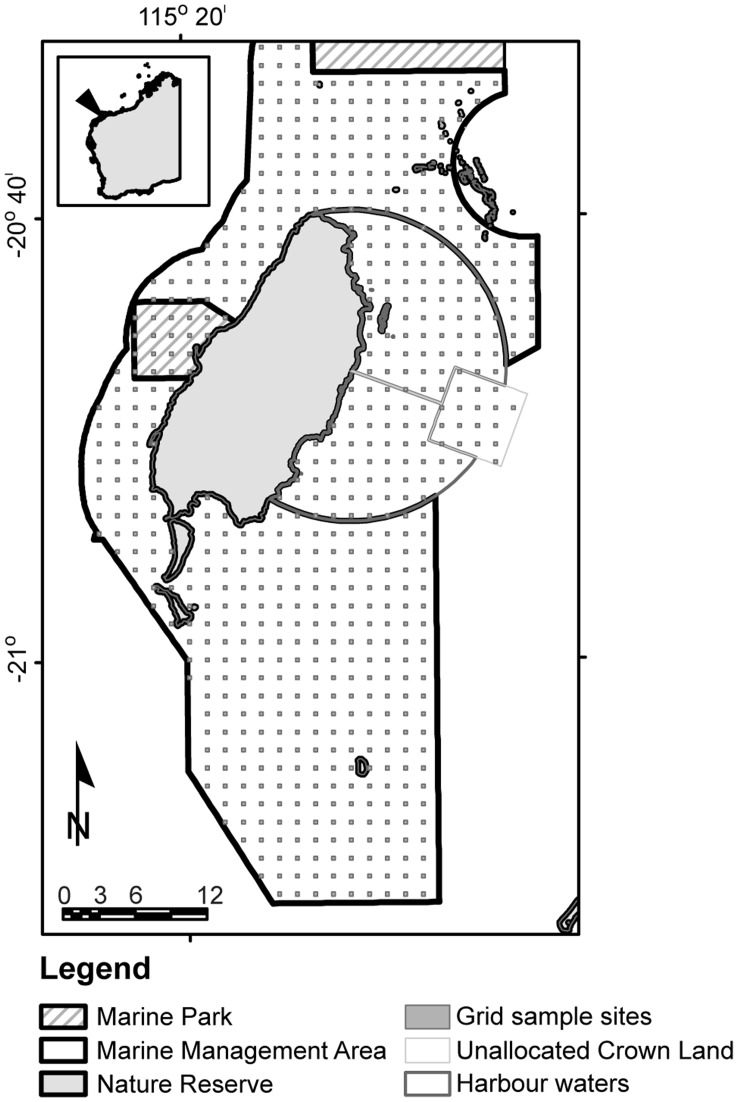
Map of the marine management areas surrounding Barrow Island and the sampling area for the grid point comparison.

**Figure 2:**
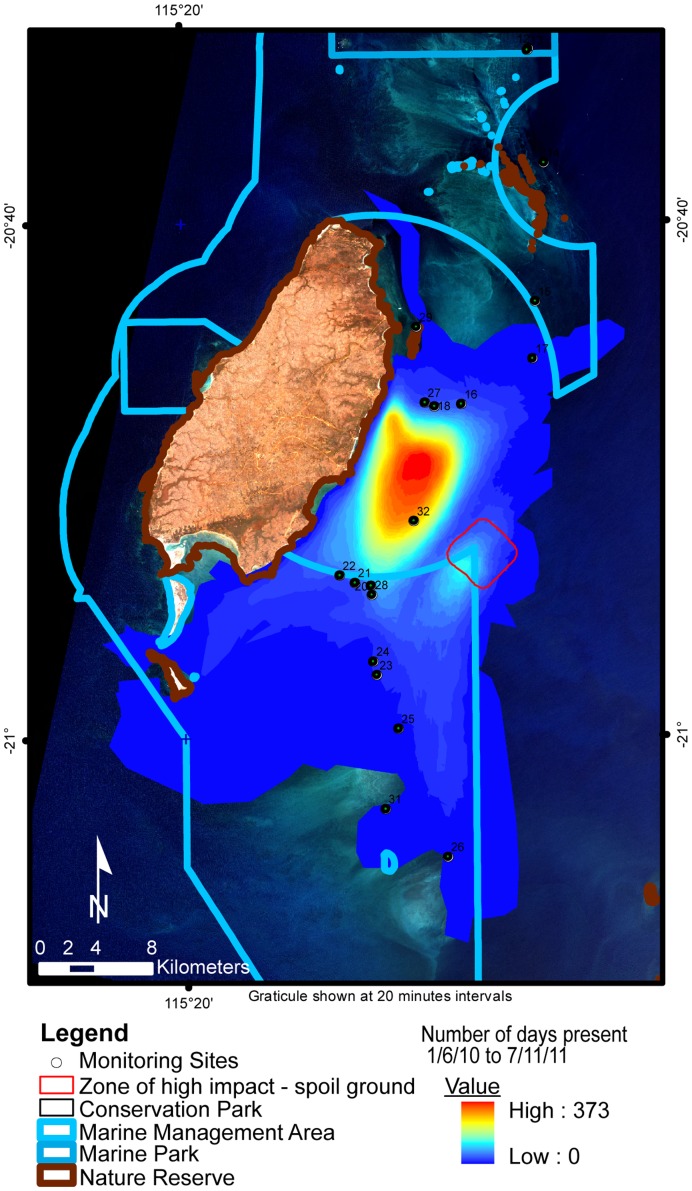
Hotspot analysis of hand digitised vectors representing the presence of dredge plume originating from the Barrow Island dredge site. Colours in the centre of the dispersal kernel represent higher numbers of days covered, decreasing to the light purple colour with low coverage. Points represent DEC monitoring sites.

The Montebello and Barrow Islands are situated in the Pilbara Offshore marine bioregions [29], approximately 1,600 km north of Perth, Western Australia, 120 km WNW of Dampier and 80 km NW of Cape Preston [30]. The waters and reefs surrounding these remote islands are characterised by geomorphological and oceanographic conditions which provide a high diversity of mainly tropical fauna including both widely distributed and endemic species [30], [31]. The marine environment is generally considered to be in a relatively undisturbed condition as a result of low human usage and strict management controls on industry activities in the area [31]. The Montebello/Barrow Islands Marine Protected Areas (MBIMPAs), incorporating the Montebello Marine Park, Barrow Island Marine Park and the Barrow Island Marine Management Area, were gazetted in 2004 and the conservation and management objectives for these reserves are expressed in the management plan for the MBIMPA [31] (Fig. 1). The Department of Environment and Conservation (DEC) monitors coral, fish, macroalgae and macro invertebrates at selected sites of significance throughout the MPAs (Fig. 2). The Gorgon Project (GP), which is based on Barrow Island (20.80°S, 115.40°E), is one of the world's largest natural gas projects and the largest single resource natural gas project in Australia's history. The GP included a dredging program that involved the removal and dumping of ∼ 7.6 million m^3^ of marine sediment over a period of approximately 18 months and started in May 2010 [32]. Modelling of the likely plume dispersal and likely impacts completed by Chevron Australia Pty Ltd prior to the commencement of dredging operations predicted the dispersal both north and south from the dredging activities dependent on the prevalent winds [32].

### Satellite Sensors

Near-daily MODIS true colour mosaic imagery (available free on the internet) provided a simple and rapid way to observe the dredge plume extent adjacent to Barrow Island. In addition, two captures of ALOS AVNIR-2 before (18 November 2006 and 23 November 2008) and one during (29 August 2010) the dredging project assisted the interpretation of the plume extent with the MODIS imagery. When available, cloud free Landsat 5 TM and 7 ETM+ images were also acquired and visually enhanced specifically for the water around Barrow Island, again to assist the interpretation of MODIS images.

Once acquired, MODIS imagery were reprojected into GDA94 MGA zone 50 and displayed in a spatial viewer with limited functionality maintained by the DEC. The dredging plume boundary was interpreted by manually digitising (drawing) a vector (digital polygon) around the plume at a scale of 1∶450000. This was found to be the optimal scale for digitising the area with 250 m by 250 m pixels resolution of MODIS. A new polygon was created for each visible plume, and then attributed with the date (Julian day) of the MODIS image being interpreted and comments. A strict file structure and naming convention was employed to aid the quick generation of “clean” datasets that were easy to display when required for quality assurance. Some MODIS images had no plumes and others had more than one plume. A year of MODIS imagery pre-dredging was also acquired and interpreted for naturally occurring plumes.

### Interpretation

The visual interpretation of daily MODIS imagery required a set of guidelines to be developed to ensure a conservative interpretation of plume boundaries. Potential guidelines include: a) comparison of imagery captured on plume-free days under similar tidal and meteorological conditions; b) only digitising plume areas that the observer had complete confidence i.e. not reef or bottom features.

Officers with little or no Geographical Information Systems (GIS) experience were trained in basic GIS skills to conduct the visual interpretation, digitising and editing of the daily plume boundary. To assist with training and quality control, the month (31 days) of October 2010 was digitised by three officers independently. The resulting boundaries were compared before the whole dataset was interpreted by one of the three observers applying strict rules developed during the one month trial.

### Plume Digitising Rules

- Three potential sources of plume were identified – Marine Offloading Facility (both extractive and land reclamation activities), turning circle (extractive activities) and the spoil ground (deposition)
- If not joined, each plume was digitised separately for each location
- Plume sources were combined in one digitised vector if the plume connects them
- All visible plumes, including both primary and subsequent plumes, were included when the observer was confident it was not substrate,
- When in doubt, areas were excluded from the polygon. Multiple images (MODIS, Landsat and ALOS) of the location without plume were used to identify natural substrate features.

### Hotspot Analysis

The definition of hotspot analysis in a GIS context was defined as ‘The highest frequency occurrence of plume coverage at a geographic position within the time period assessed [34]. A hotspot analysis was run on the cumulative daily digitised plume boundaries to provide a dataset describing the number of days the plume was present at any position within the Barrow Island Marine Park and surrounds. This involved appending the datasets in ArcGIS [35] and determining the frequency of plume presence (days) in IDRISI [36] for the entire period of the dredge operations (525 days). DEC monitoring sites were buffered by 100 m and intersected with the resulting frequency dataset for the entire dredge period to extract the frequency of days each site was under the influence of the plume. This method was also used to determine the amount of time the plume covered the Montebello and Barrow Islands Marine Protected Areas. This was calculated by determining the area of plume that overlapped the boundary of the Barrow Island Marine Management Area to the north and south of the Barrow Island Port boundary.

### Total Suspended Solids

Seawater samples were collected for 13 sites, each in clean 1 L bottles. The GPS coordinates of each sample site were also recorded. On return to shore, TSS samples were filtered under low vacuum onto dry, pre-weighed Whatman GF/F filters (47 mm diameter) with a nominal pore size of 0.7 µm. Filters were dried for 24 hours and subsequently weighed to determine the TSS load in units of mg L^−1^.

### Above-water Radiometry

Above-water hyperspectral radiometric data were collected using a DALEC three-channel radiometer developed at Curtin University and available from In-situ Marine Optics (http://insitumarineoptics.com/). The DALEC instrument measures reflectance properties in a similar fashion to satellite-borne sensors. However, the DALEC is under-atmosphere and thus, atmospheric corrections do not have to be considered when processing the data. The DALEC sensor collects radiometric data over the spectral range 400–900 nm with a nominal resolution of 3 nm. The DALEC continually logs radiometric data and GPS coordinates so that locations of interest can be identified and processed. DALEC hyperspectral data were convolved with MODIS spectral response functions to give under-atmosphere remote sensing reflectance, *R_rs_*, data with the same spectral resolution and band response as the MODIS sensor.

### TSS Model Development

**Figure 3:**
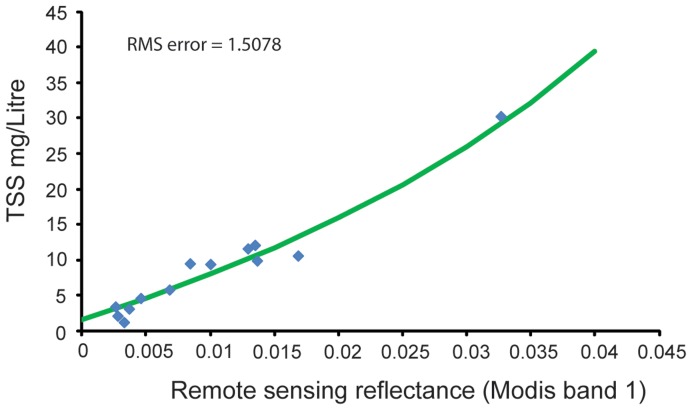
Plot of observed TSS relative to DALEC-synthesised band 1 reflectance (blue diamonds) from waters adjacent to Barrow Island. The TSS model is shown fitted through the observed data (green line).

Using the measured TSS from the filtered water and the coincident DALEC *R_rs_* data, an empirical algorithm was developed which was then applied to MODIS imagery. A non-linear least squares fitting algorithm was used to develop a model which relates TSS concentration to at-surface DALEC-synthesised MODIS band 1 reflectances (See Figure S1). The model fitted through the observed data is shown in Figure 3 (r-squared  = 0.959).

### MODIS TSS Algorithm

On the 9^th^ August, 2011 a field campaign (according to DEC permit numbers SC001263 & SW013765) was undertaken at Barrow Island during which a total of 13 sites were sampled. For each site, at-surface radiometric data were collected with coincident TSS data. These in-situ data were used to develop a regional algorithm for determining TSS concentration from MODIS data for the Barrow Island dredge plume using the processes describe herein. MODIS data were accessed from the West Australian Satellite Technology and Applications Consortium (WASTAC) archive (http://www.wastac.wa.gov.au/) over a geographic domain containing Barrow Island. MODIS data were processed using the SeaWiFS Data Analysis System (SeaDAS) [37] to produce atmospherically corrected band 1 (645 nm) remote sensing reflectance. The algorithm in equation (1.) with the regionally fitted coefficients was applied to the MODIS *R_rs_* data on a pixel-by-pixel basis to derive the L2 MODIS TSS product. During processing, nuisance/bad pixels containing land and/or clouds were masked out. No attempt to account for shallow water reflectance was incorporated into the processing. These data were then georeferenced and regridded before the region surrounding Barrow Island was extracted for this comparison.

### Comparison of Total Suspended Sediment Data with the Results of the Digitised Method

TSS images were only compared with hand digitised MODIS true colour images if they had no distortion due to low view angle, were cloud free and provided full images covering the area of interest. The comparison used a grid sampling method of 623 sites covering the Barrow Island Marine Management Area and the Barrow Port Area (Fig. 1). Both datasets were intersected with the grid points to return a binary value for each point to represent presence (1) or absence (0) of the plume in the digitised and TSS dataset. The TSS data were then converted to binary data for 6 thresholds of TSS, >1 mg L^−1^, >2 mg L^−1^, >3 mg L^−1^, >4 mg L^−1^, >5 mg L^−1^, >6 mg L^−1^ so both the digitised data and the TSS data samples could be directly compared as presence/absence data. Comparisons of digitised data to the range of TSS values were analysed using a one-way analysis of similarities (ANOSIM) in PRIMER [38] to determine the R statistic and any significant differences between the data sets. The R statistic is a useful tool to compare the degree of separation of two data sets and is as important (if not more so) as the statistical significance [39]. The R statistic in ANOSIM provides a result between 1 and −1, results closer to zero suggest more similarity between two data sets [38], [39]. The data sets were also represented in a non-metric Multidimensional Scaling (MDS) ordination plot to visualize the differences between the digitising and each of the TSS data sets. The non-metric MDS is a visual representation of the similarity of more than one data set based on the chosen factors. There is no metric to the axes and hence no labeling. In this study, the factors are the presence/absence of plume recorded using hand digitizing and several levels of TSS measurement. Points become closer the more similar they are. The more dispersed the points become the more dissimilar are the shapes of the plumes over the grid points in Figure 1 (See [39] for more detail).

## Results

### Image Interpretation

Three observers digitised one month of daily true colour MODIS imagery captured during the dredging operation. Inter-observer variability was not significantly different (p  = 0.647) and resulted in an average of 53 km^2^ area of plume digitised with an average standard deviation (SD) of 10 km^2^ (18%), a minimum SD of 0.4 km^2^ (0.7%) and a maximum SD of 36 km^2^ (68%). The main difference between interpreters was the level of conservatism surrounding what was regarded as either concentrated or marginal plume (areas where the digitiser was unsure of the plumes presence). Observers attempted to be conservative in their interpretation, but some were more so than others. For example, often conservative observers digitised the concentrated plume and not the areas of marginal plume (areas that were questionably plume or reef). A set of rules were generated based on the inter-observer comparison to improve consistency and confidence of the sole observer who digitised the entire dredge period (approximately 18 months).

### Plume Monitoring

The plume was monitored using satellite MODIS imagery from 19 May 2010 to 7 November 2011 for the entirety of the dredging operations resulting in 538 possible days where the plume could be observed and digitised. It should be noted that no plume was observed until 1 June 2010. Both MODIS Aqua and Terra satellite imagery were employed for interpretation, with the choice of each dependent on image quality. Vectors were not digitised for 24% (127 days) of the total dredge period due to cloud cover and poor satellite image coverage (i.e. the sensor did not capture the Barrow Island region of interest). The number of days individual DEC in-situ monitoring sites were covered by the plume ranged from <1% to 70% of the days digitised (Fig. 2).

**Figure 4:**
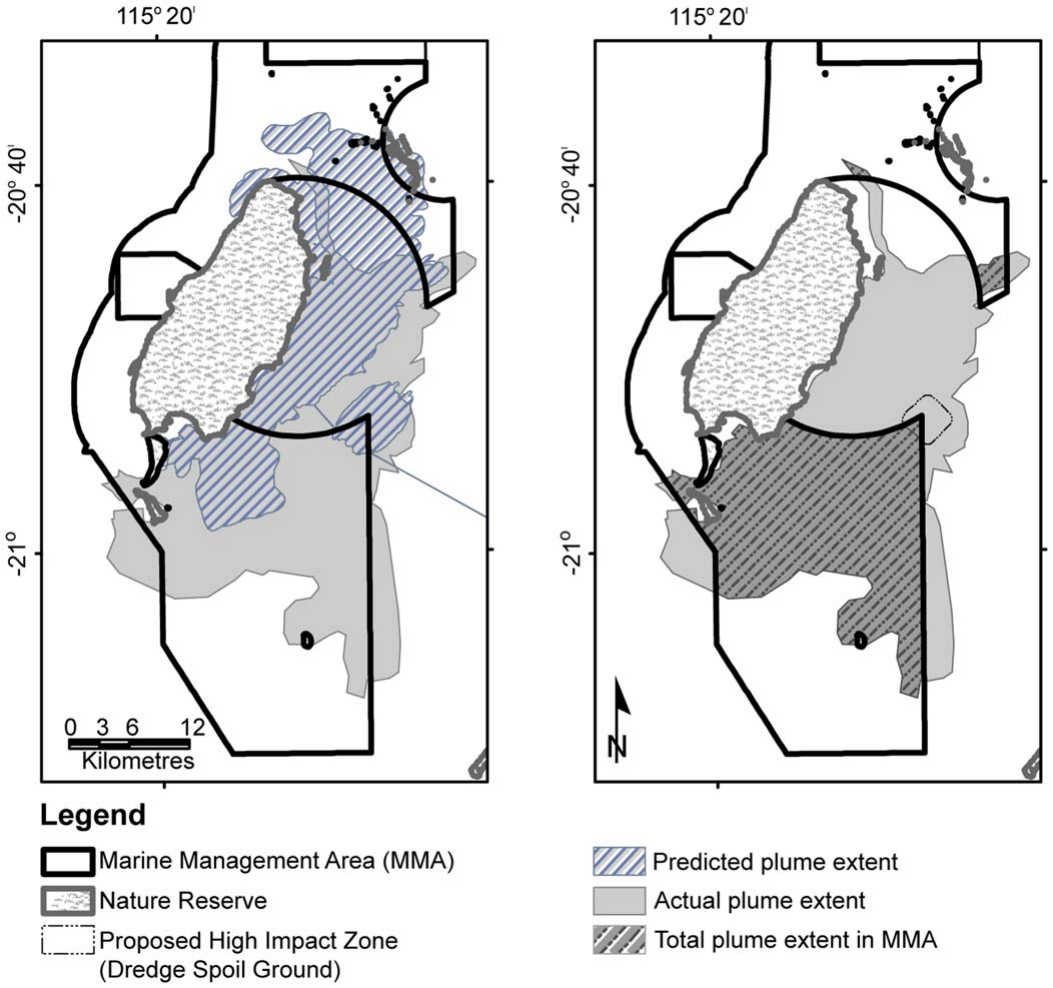
Map showing Montebello and Barrow Island Protected Areas, and a) the extent of the modelled plume and the actual dredge plume extent as defined by hand digitising MODIS images; and b) The overlap of the dredge plume into the Barrow Island Marine management area, over the duration of dredge period as defined by hand digitising MODIS images.

Plume dispersal modelling completed by Chevron Australia Pty Ltd, prior to the commencement of dredging operations predicted coverage of 465 km^2^ moving both north and south from the dredging activities dependent on the prevalent winds [33]. Our study found that the plume moved predominantly southward with minimal days of northward movement. Plume digitising showed that 279 km^2^ overlapped with the modelling and 455 km^2^ did not (Fig. 4A). A hotspot analysis was undertaken to highlight how often sites of high biological significance were covered by the plume. Individual DEC coral reef monitoring sites were covered from 1 to 296 of the 411 days observed (Fig. 2). During the period of the dredge operation, the cumulative plume coverage over the marine management area (MMA) reached 395 km^2^, and the total MMA within the expected high impact area of the spoil ground was 2.3 km^2^ (see Fig. 4B).

### Hand Digitising vs Remote Sensing TSS Comparison

**Figure 5:**
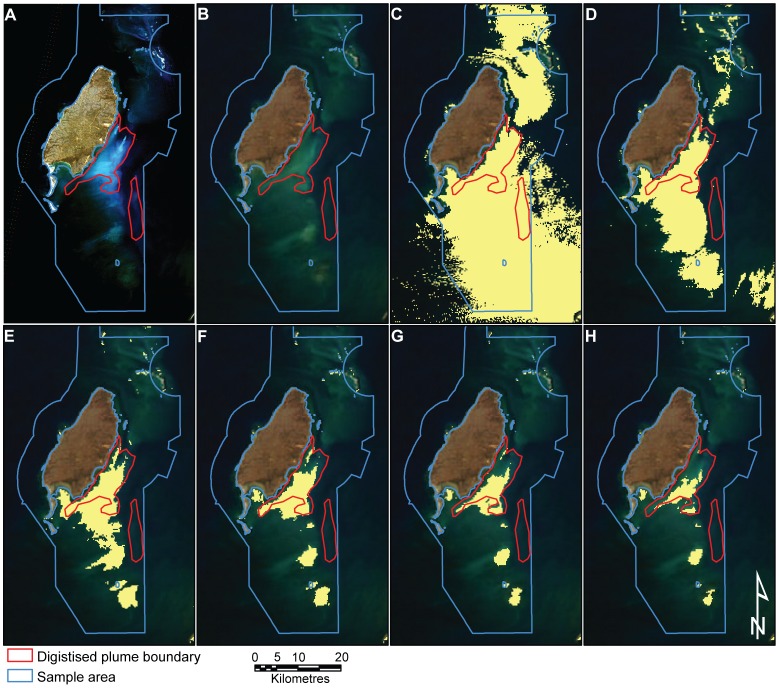
Series of images showing how the digitised plume of a MODIS image relates to a clearer high resolution image and the range of TSS thresholds analysed in this study. A) Landsat image B); MODIS digitised interpretation; C) TSS threshold at >1 mg L^−1^; D) TSS threshold at >2 mg L^−1^; E) TSS thresholded at >3 mg L^−1^; F) TSS threshold at >4 mg L^−1^; G) TSS threshold at >5 mg L^−1^; H) TSS threshold at >6 mg L^−1^. Red line is the outline of the hand digitised plume from the MODIS imagery. Purple Line is the Barrow Island Marine Management Area and includes the Barrow Port area.

**Figure 6:**
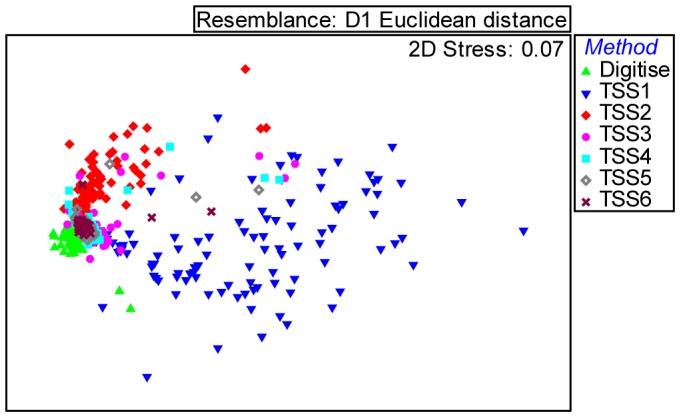
Non-metric Multidimensional scaling plot showing the similarity of points representing the spatial distribution of the dredge plume per day for hand digitised plume vectors and six levels of TSS measured from MODIS imagery.

Hand digitising (MODIS ONLY) enabled interpretation of 304 days of useful imagery compared to 116 days derived from processing MODIS data to a TSS product that was suitable to interpret clearly with full coverage, free of cloud and distortion due to low viewing angles. The analysis to compare the results of both methods was only carried out for days that both methods were able to interpret the MODIS data. The approach adopted was to compare the spatial extent of the plume derived by each method. The delineation of plume extent based on the remote sensing TSS approach depends upon selection of the TSS threshold. For this work we compared images based on hand digitizing and remote sensing TSS with TSS thresholds of 1, 2, 3, 4, 5 and 6 mg L^−1^ (See Fig. 5). Each point in the MDS plot represents the spatial distribution of grid points covered by the plume on a day (Fig. 6). The MDS plot represents the similarity of plume presence detected by the methods for each day. The very low stress (0.07) indicates that the data were well represented in 2 dimensions. The MDS illustrates a cluster of points in the left of the panel which represents the days when each of the methods had a similar result. This was likely to represent the primary main dredge plume area. However, consistent differences were found as there were no complete overlaps between the digitising of the plume and any of the different TSS concentration threshold extents. The widespread TSS points throughout the rest of the panel suggest highly variable plume spatial extents at different TSS thresholds. The high correlation (by visual inspection) of the location of TSS features and the locations of shallow bathymetric features suggests that the high variability in plume extent is probably due to days where the shallow benthic features were incorrectly interpreted as plume. This is particularly the case on strong wind days and after large rain periods with increased river outflow and natural turbidity moving from inshore areas to the Southern Barrow Shoals. It is important to note that image quality plays a big part in the amount of shallow water benthic habitat observed using TSS. Image quality can be affected by satellite angle, sun angle, tides and wind energy.

The results of the ANOSIM show that TSS 3 mg L^−1^ is most similar to the hand digitised method (Table 1). However, the differences between TSS 2 to 4 mg L^−1^ are so slight that either of these results may be equally representative depending on the day analysed. For example, TSS 4 mg L^−1^ is probably the most similar based on the visual representation of Julian day 205 (24 July) 2010 (Fig. 5). There is, however, no exact visual matchup, which arises from the lack of any TSS reading of the plume from the spoil ground (except TSS1) and the inclusion of benthic habitats (particularly areas of sand) in shallow areas of the Southern Barrow Shoals at all TSS levels. TSS1 consistently overestimated the dredge plume and TSS 5 and 6 underestimated the plume near the dredge location. This highlights the issues of determining spatial extent of dredge plumes in clear water shallow environments with TSS.

**Table 1:** The R statistic shows the similarity of the spatial distribution of plume estimated by a range of Total Suspended Sediment values compared to hand digitised vectors of dredge plume due to the Barrow Island dredge operations.

TSS	1	2	3	4	5	6	7	8	9
**R Statistic**	0.539	0.484	0.439	0.484	0.527	0.539	0.545	0.554	0.555

The lowest R statistic (most similar) is underlined.

## Discussion

Shallow water benthic communities suffer from both loss of light and smothering from sediments derived from dredging activities. Quantifying the extent to which an area is impacted by a dredge plume is critical to attributing changes detected to the benthic community from these events. In this paper we have shown that the hand digitising approach described is a useful tool for monitoring dredge plume movements in shallow coral reef environments (Table 2). Using visual inspection of MODIS images to digitise the plume one can discern, with a reasonable level of certainty, the differences between sediment plumes generated by dredging activities from natural features such as shallow water habitats and/or wave and wind generated resuspension of sediments. Through the use of reference images, including high resolution satellite imagery (i.e. Landsat and ALOS imagery), topographic maps and aerial photography [26], [32] the image digitiser can distinguish the boundaries of dredge generated plume event from these natural features. Further confirmation of the plume can always be achieved through the incorporation of in-situ light and turbidity loggers and sediment traps where available.

**Table 2:** Summary of the two methods used to characterize the extent of a dredge plume at Barrow Island, NW Australia.

	Digitising	TSS
Information Provided	Temporal coverage in a spatial context	Estimate of concentration temporally and spatially
Staff training	Basic	Professional expertise: University or technical remote sensing
Ability to differentiate shallow benthos from plume	Relatively accurate with some site familiarity. Can be conservative.	Without Mask/threshold: overestimate dredge plume; With Mask/threshold: Potential to underestimate dredge plume.
Time	1–2 weeks to digitize a whole year	Developing method takes time. Once process is working and running, results could be near real time
Fieldwork requirement	Not essential but helpful to validate observations.	Ground truthing results derived from an algorithm is essential. Can require extensive costly fieldwork.
Arbitrariness	High	Nil
Ability to interpret	High	Moderate
Reprocessing	Slow	Rapid

MODIS images are captured at a relatively low resolution of 250 m×250 m (compared to ALOS at 10 m) sometimes providing images of poorer quality. However, twice daily passes of the MODIS satellite over the study area increased the opportunity to capture at least one good quality daily image. While poorer images were often un-useable with the TSS method, the hand digitising method had a greater chance of using these images through the comparison with other pre-dredge MODIS and higher resolution images that assisted the interpreter. This resulted in a greater number of days interpreted with the hand digitising methods compared with the semi-automated TSS method (304 hand digitized versus 116 TSS of the total 411 days captured). Furthermore, the plume area was limited to a relatively small area around Barrow Island requiring only 1 to 3 polygons digitised per day. Focusing on a small area of potential plume extent allowed the interpreter to quickly become familiar with the appearance of natural benthic features in the imagery at times with or without dredge plume and under different weather conditions. This is in stark contrast to the extent and number of units processed during fire digitising which is done within Australia on a regular basis [21]. Therefore, a digitised visual interpretation of the plume in MODIS imagery was a time efficient option.

This study has shown that once people are trained to interpret MODIS imagery within an ecological and environmental context, within the limitations of the image quality, the confidence in the human interpretation of a boundary was greater than the semi-automated remote sensing approach [40]. The dredge plume hand digitisation methodology developed for this project was found to be time efficient for an inexperienced GIS user recently trained in the method. In comparison, remote sensing TSS algorithm approaches use techniques that require specialised skills to develop the regionally tuned models and to produce code to process satellite data with the models. The development of regionally tuned models requires fieldwork, which can be costly and time consuming. The models are tuned to the constituents of suspended sediment and if these change due to the stratification of the sea floor then this would affect the accuracy of the TSS estimates from MODIS data. Application of these procedures in an automated computer processing environment provides the advantage of rapid processing time, and enables faster delivery of products. Developing such a system however requires significant technical expertise and may be outside the financial capacity of many government and non-government organisations.

At present, the semi-automated TSS approach has difficulty discerning between shallow reef habitat and dredge plume (Fig. 5). This can be improved by either masking out (removing or ignoring) any shallow water environments or thresholding (reducing the sensitivity) the level of TSS detected by the algorithm. However, both these methods limit the ability to detect plume coverage over the shallow areas. Statistically, there is an apparent similarity between the hand digitistation and semi-automated TSS methods at TSS threshold values from 2 to 4 mg L^−1^ using ANOSIM, however, overlaying hand digitised vectors with TSS thresholded images (Fig. 5) showed that separating the dredge plume from shallow benthic features was something that the eye could achieve more consistently than a semi-automated algorithm. This is highlighted in the example provided (Fig. 5). Removing shallow areas from the analysis would provide false negatives, while hand digitsing allows the interpretation of the plume over the shallow benthic environments. Improvements to differentiate shallow water habitats are in development to counter this issue (unpublished: McKinna), however the rapid semi-automated method will remain limited in relation to this inaccuracy and would become time consuming to correct with visual inspection of daily MODIS imagery to determine if there is plume crossing the reef. This is similar for hand digitizing, but organizations do not require highly trained and specialized staff to complete such an approach.

The benefit of the remote sensing TSS algorithm approach is that it offers a consistent quantitative estimate of sediment concentration and therefore gives an indication of potential impact a dredge plume may have on benthic organisms such as coral communities in the Barrow Island region. Hand digitising does not provide concentration estimates and can be used only as a spatial recognition tool for “snapshot monitoring”. However, temporal snapshot monitoring provides invaluable information for managers or researchers to understand the extent of plume coverage over significant sites, such as coral monitoring sites, fish aggregation spawning sites or marine park boundaries. Another advantage of the TSS approach is the ability to perform re-processing of the entire MODIS imagery archive if improvements to the technique are made in the future. This processing can be run on a high performance computing system allowing an entire 10-year archive to be processed in a little over a week.

### Conclusion

Ultimately the improvement of the semi-automated method for the process of extracting the plume boundaries would be ideal, however, with the tools presently available, the hand digitizing technique provides some important benefits to the monitoring of dredge plume events particularly in relation to monitoring shallow benthic communities. Not only has this project shown that hand digitising of dredge plumes, particularly in shallow water environments, is a useful tool, it has also provided a dataset that can be used to improve future dredge plume modelling in the region and assist as a reference dataset to improve automated remote sensing methods in the future. This research has demonstrated that sometimes the simplest methods can provide time and cost effective information to help manage impacts to our natural environment. Furthermore, it highlights the need to improve semi-automated processes in this field.

## Supporting Information

Figure S1 **The model used to relate TSS to MODIS band 1 reflectance,** ***R_rs_(b1)*** **, where,** ***a_w_*** **and** ***b_bw_*** **are the spectral absorption and scattering properties of pure water respectively.** The coefficients c_0_, c_1_, and c_2_ are constants with values of 0.1172490, 0.00479719, and −0.00629920 respectively.
(GIF)
