# Supplementary figures and images for: Digitise This! A Quick and Easy Remote Sensing Method to Monitor the Daily Extent of Dredge Plumes
Source: PLoS One. 2012 Dec 11;7(12):e51668. doi: 10.1371/journal.pone.0051668 (PMC3519868; doi:10.1371/journal.pone.0051668)

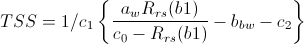

Supplement: Figure S1 — The model used to relate TSS to MODIS band 1 reflectance, Rrs(b1) , where, aw and bbw are the spectral absorption and scattering properties of pure water respectively. The coefficients c0, c1, and c2 are constants with values of 0.1172490, 0.00479719, and −0.00629920 respectively. (GIF) [file pone.0051668.s001.gif]
